# Imaging the vessel wall in major peripheral arteries using susceptibility weighted imaging: visualizing calcifications

**DOI:** 10.1186/1532-429X-11-S1-O12

**Published:** 2009-01-28

**Authors:** Qi Yang, Kuncheng Li, Jiangtao Liu, S Barnes, Z Wu, J Neelavalli, J Hu, EM Haacke

**Affiliations:** 1 Xuanwu Hospital, Beijing, PR China; 2 Wayne State University, Detroit, MI USA; 3 McMaster University, Hamilton, ON Canada

**Keywords:** Vessel Wall, Arterial Wall, Phase Image, Popliteal Artery, Calcification Area

## Introduction

Magnetic resonance imaging (MRI) has been used for many years to study atherosclerosis [1]. Black blood techniques are the most ubiquitous and are used to suppress the signal from flowing blood, making the vessel wall more conspicuous. The purpose of this study was to demonstrate a novel approach to imaging the vessel wall and vessel wall calcification using susceptibility weighted imaging [2] (SWI) with no need to suppress the signal from the blood.

## Methods

### Optimizing the imaging parameters

The SWI sequence parameters were optimized to allow for the best visualization of the femoral artery lumen in the magnitude images and the arterial wall in the phase images. Parameters such as resolution (for time considerations), flip angle (for contrast in the magnitude images) and echo time (for phase contrast) were considered.

### Vessel wall magnitude and phase measurements

ROIs from the top to the bottom of the visible portions of the femoral artery were taken. The lumen SNR and muscle SNR were calculated on both magnitude and phase images. The contrast-to-noise ratio of vessel wall/lumen and vessel wall/muscle was also calculated.

### Patients study

A series of 18 subjects were imaged with multi-detector computed tomography (MDCT) and high resolution susceptibility weighted imaging (SWI) at 3 T.

### Calcification Measurements

The area of calcification was manually measured on CT images and MR images (both magnitude and phase images) by an experienced radiologist. SPIN software (Detroit, MI) was used to interpolate the images by a factor of 4 and measure the calcifications. The correlation of calcification area (CA) between CT and MR images was performed and a Pearson correlation coefficient calculated. The agreement of CA measurements by MR and CT was assessed by using the Bland and Altman plot.

## Results

**Figure 1:**
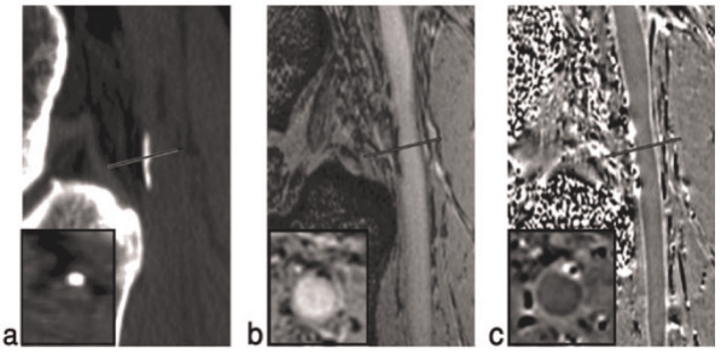
**(a) CT scan showing calcification at the edge of the popliteal artery just behind the knee**. (b) Magniture gradient echo image showing the signal loss from the calcification of the same area. (c) Phase image showing the diamagnetic effect from the calcification. Note the simliar shape and extent of the calcification in both the CT and MR results. Inserts are zoomed images of the cross-section of the vessels in reformatted transverse images.

The optimal choice of imaging parameters was found to be: TE = 15.6 ms (in-phase for fat); TR = 25 ms, FA = 10°, BW = 80 Hz/pixel, resolution = 0.5 mm × 0.5 mm in-plane and 1.0 mm through-plane, with an acquisition matrix of 512 × 384 × 64 (for read, phase and slice-select direction) and a total scan time of 8 minutes. The magnitude contrast-to-noise ratio (CNR) between artery and vessel wall was 12:1. The phase CNR between the arterial wall and the lumen was 7:1. A total of 19 calcifications in the femoral vessel wall were identified with SWI in 8 subjects. The mean area of calcification measured on CT, magnitude and phase images was 0.37 ± 0.17 cm^2^, 0.29 ± 0.13 cm^2^, 0.38 ± 0.18 cm^2^ respectively. The Pearson correlation coefficient of the measured lesion area between CT and magnitude image is 0.85 (p < 0.001); between CT and phase image is 0.92 (p < 0.001). A typical case having popliteal artery calcification is shown in Figure 1. Both magnitude and phase images show the calcifications clearly in the popliteal artery wall and correlate well with the CT image.

## Conclusion

SWI offers a means to image a large field-of-view over which the arterial wall can be clearly seen in both magnitude and SWI filtered phase images. These lesions were seen in CT and SWI and correlated well in both size and position with both methods. We anticipate that SWI will play a complementary role to the current multi-contrast approach in studying atherosclerosis.
